# Supplementary material for: Designed mosaic nanoparticles enhance cross-reactive immune responses in mice
Source: Cell. 2025 Feb 20;188(4):1036–1050.e11. doi: 10.1016/j.cell.2024.12.015 (PMC11845252; doi:10.1016/j.cell.2024.12.015)
Supplement: Document S1. Tables S1–S4 and supplemental references [file mmc1.pdf]

Cell, Volume *188*

Supplemental information

Designed mosaic nanoparticles enhance cross-reactive immune responses in mice

Eric Wang, Alexander A. Cohen, Luis F. Caldera, Jennifer R. Keeffe, Annie V. Rorick, Yusuf M. Adia, Priyanthi N.P. Gnanapragasam, Pamela J. Bjorkman, and Arup K. Chakraborty

Supplementary Information

Designed mosaic nanoparticles enhance cross-reactive immune responses in mice

Eric Wang[1,8], Alexander A. Cohen[2,8], Luis F. Caldera[2,8], Jennifer R. Keeffe[2], Annie V. Rorick[2], Yusuf M. Adia[2,6], Priyanthi N.P. Gnanapragasam[2], Pamela J. Bjorkman[2,*], and Arup K. Chakraborty[1,3-5,7,9,*]

[1]Institute for Medical Engineering and Science, Massachusetts Institute of Technology, Cambridge, MA 02139.

[2]Division of Biology and Biological Engineering[2], California Institute of Technology, Pasadena, CA 91125.

[3]Department of Chemical Engineering, Massachusetts Institute of Technology, Cambridge, MA 02139.

[4]Department of Physics, Massachusetts Institute of Technology, Cambridge, MA 02139.

[5]Department of Chemistry, Massachusetts Institute of Technology, Cambridge, MA 02139.

Present address: [6]School of Clinical Medicine, University of Cambridge, Hills Rd, Cambridge, CB2 0SP, UK

[7]Ragon Institute of Massachusetts General Hospital, Massachusetts Institute of Technology, and Harvard University, Cambridge, MA 02139.

[8]These authors contributed equally.

[9]Lead contact

*Correspondence: arupc@mit.edu; bjorkman@caltech.edu

Table S1. **Antibodies used to calculated escape, related to Figure 1.** Antibodies in the deep mutational scanning data[1-5] used to calculate escape.

Antibody	RBD epitope class[6]
COV2-2196	class 1
S2H14	class 1
COV2-2165	class 1
COV2-2832	class 1
S2E12	class 1
LY-CoV016	class 1
REGN10933	class 1
C105	class 1
S2X58	class 2
S2D106	class 2
S2H13	class 2
S2H58	class 2
S2X16	class 2
LY-CoV555	class 2
C121	class 2
C144	class 2
C002	class 2
COV2-2096	class 2
COV2-2050	class 2
COV2-2479	class 2
C135	class 3
S2X227	class 3
COV2-2499	class 3
REGN10987	class 3
S309	class 3
COV2-2130	class 3
C110	class 3
CR3022	class 4
COV2-2677	class 4
S2X35	class 4
COV2-2094	class 4
S304	class 4
S2H97	class 4
COV2-2082	class 4
S2X259	class 4

Table S2. **Mutations used to generate new RBDs, related to Figure 1.** The 20 RBD positions with highest escapes from class 1 and 2 anti-RBD antibodies[6] based on DMS data[1-5] and their assigned amino acid. An assigned amino acid was selected as the amino acid with the largest mean escape fraction relative to the WA1 amino acid, which also was not a charged-to-hydrophobic mutation.

Antibody RBD-epitope class	RBD position with high escape	Assigned amino acid
1	417	Y
1	420	Q
1	449	D
1	455	R
1	456	G
1	460	L
1	472	P
1	473	F
1	475	N
1	476	S
1	484	K
1	485	S
1	486	A
1	487	K
1	489	L
1	496	V
1	498	D
1	500	Q
1	501	A
1	504	L
2	346	D
2	447	D
2	449	K
2	450	T
2	452	R
2	455	G
2	456	A
2	472	P
2	473	F
2	475	N
2	481	K
2	483	R
2	484	R
2	485	K
2	486	P
2	487	K
2	489	V
2	490	K
2	493	K
2	494	R

Table S3. Mutations relative to the WA1 RBD for RBDs used to construct mosaic-2$_{COMs}$ and mosaic-5$_{COM}$, related to Figure 1. Designed RBDs with their specific mutations relative to the SARS-CoV-2 WA1 RBD. 3 mutations are in RBD positions with high escape against class 1 antibodies[6] based on DMS data[1-5] (class 1 escape mutations), and 3 mutations are in RBD positions with high escape against class 2 antibodies (class 2 escape mutations). RBDs that pass experimental validation in terms of expression (Figure 3B) and binding to class 3 and 4 antibodies but not class 1 and 2 antibodies (Figure 3D) are bolded.

RBD Pair	RBD ID	Class 1 escape mutations	Class 2 escape mutations
1	**RBD1**	L455R, F486A, N487K	E484R, Y489V, F490K
	**RBD2**	K417Y, A475N, Q498D	G447D, L452R, Q493K
2	RBD3	L455R, F456G, N487K	E484R, F490K, Q493K
	**RBD4**	A475N, Y489L, N501A	Y449K, L452R, F486P
3	**RBD5**	K417Y, F486A, Q498D	F456A, E484R, S494R
	**RBD6**	L455R, A475N, N487K	I472P, F490K, Q493K
4	**RBD7**	A475N, F486A, N487K	L452R, E484R, Y489V
	RBD8	L455R, F456G, Y473F	G485K, F490K, Q493K
5	**RBD9**	F486A, N487K, Y489L	Y449K, L452R, E484R
	**RBD10**	L455R, I472P, A475N	F456A, F490K, S494R

Table S4. Sarbecovirus RBDs used to construct mosaic-7$_{COM}$, related to Figure 1. Selected sarbecovirus RBDs along with their GenBank[7] accession numbers, chosen residue numbers based on alignment with the SARS-CoV-2 WA1 RBD, and clade. RBDs that passed experimental validation in terms of expression (Figure 3B) and binding to class 3 and 4 antibodies but not class 1 and 2 antibodies (Figure 3D) are bolded. The clade is defined as described in Starr et al.[8]

Virus	Accession	Residue number	Clade
**LYRa3**	AHX37569.1	310-527	1a
**Khosta-2**	QVN46569.1	307-522	3
**C028**	AAV98001.1	306-523	1a
**SHC014**	QJE50589.1	307-524	1a
**BM48-31**	YP 003858584.1	310-524	3
**BtKY72**	APO40579.1	309-526	3
**pang17**	QIQ54048.1	317-549	1b
RaTG13	QHR63300.2	319-541	1b

References

1. Dong, J., Zost, S.J., Greaney, A.J., Starr, T.N., Dingens, A.S., Chen, E.C., Chen, R.E., Case, J.B., Sutton, R.E., Gilchuk, P., et al. (2021). Genetic and structural basis for SARS-CoV-2 variant neutralization by a two-antibody cocktail. Nature Microbiology *6*, 1233-1244. 10.1038/s41564-021-00972-2.
2. Greaney, A.J., Starr, T.N., Gilchuk, P., Zost, S.J., Binshtein, E., Loes, A.N., Hilton, S.K., Huddleston, J., Eguia, R., Crawford, K.H.D., et al. (2021). Complete Mapping of Mutations to the SARS-CoV-2 Spike Receptor-Binding Domain that Escape Antibody Recognition. Cell Host & Microbe *29*, 44-57.e49. 10.1016/j.chom.2020.11.007.
3. Greaney, A.J., Starr, T.N., Barnes, C.O., Weisblum, Y., Schmidt, F., Caskey, M., Gaebler, C., Cho, A., Agudelo, M., Finkin, S., et al. (2021). Mapping mutations to the SARS-CoV-2 RBD that escape binding by different classes of antibodies. Nature Communications *12*, 4196. 10.1038/s41467-021-24435-8.
4. Starr, T.N., Greaney, A.J., Dingens, A.S., and Bloom, J.D. (2021). Complete map of SARS-CoV-2 RBD mutations that escape the monoclonal antibody LY-CoV555 and its cocktail with LY-CoV016. Cell Reports Medicine *2*, 100255. 10.1016/j.xcrm.2021.100255.
5. Starr, T.N., Czudnochowski, N., Liu, Z., Zatta, F., Park, Y.-J., Addetia, A., Pinto, D., Beltramello, M., Hernandez, P., Greaney, A.J., et al. (2021). SARS-CoV-2 RBD antibodies that maximize breadth and resistance to escape. Nature *597*, 97-102. 10.1038/s41586-021-03807-6.
6. Barnes, C.O., Jette, C.A., Abernathy, M.E., Dam, K.-M.A., Esswein, S.R., Gristick, H.B., Malyutin, A.G., Sharaf, N.G., Huey-Tubman, K.E., Lee, Y.E., et al. (2020). SARS-CoV-2 neutralizing antibody structures inform therapeutic strategies. Nature *588*, 682-687. 10.1038/s41586-020-2852-1.
7. Benson, D.A., Cavanaugh, M., Clark, K., Karsch-Mizrachi, I., Lipman, D.J., Ostell, J., and Sayers, E.W. (2013). GenBank. Nucleic Acids Res *41*, D36-42. 10.1093/nar/gks1195.
8. Starr, T.N., Zepeda, S.K., Walls, A.C., Greaney, A.J., Alkhovsky, S., Veesler, D., and Bloom, J.D. (2022). ACE2 binding is an ancestral and evolvable trait of sarbecoviruses. Nature *603*, 913-918. 10.1038/s41586-022-04464-z.
